# Nomogram for predicting disseminated intravascular coagulation in heatstroke patients: A 10 years retrospective study

**DOI:** 10.3389/fmed.2023.1150623

**Published:** 2023-03-15

**Authors:** Qingbo Zeng, Lincui Zhong, Nianqing Zhang, Longping He, Qingwei Lin, Jingchun Song

**Affiliations:** ^1^Intensive Care Unit, The 908th Hospital of Logistic Support Force, Nanchang, China; ^2^Intensive Care Unit, Nanchang Hongdu Hospital of Traditional Chinese Medicine, Nanchang, China

**Keywords:** heatstroke, disseminated intravascular coagulation, predictor, nomogram, thromboelastography

## Abstract

**Background:**

Disseminated intravascular coagulation (DIC) can lead to multiple organ failure and death in patients with heatstroke. This study aimed to identify independent risk factors of DIC and construct a predictive model for clinical application.

**Methods:**

This retrospective study included 87 patients with heatstroke who were treated in the intensive care unit of our hospital from May 2012 to October 2022. Patients were divided into those with DIC (*n* = 23) or without DIC (*n* = 64). Clinical and hematological factors associated with DIC were identified using a random forest model, least absolute shrinkage and selection operator (LASSO) regression and support vector machine-recursive feature elimination (SVM-RFE). Overlapping factors were used to develop a nomogram model, which was diagnostically validated. Survival at 30 days after admission was compared between patients with or without DIC using Kaplan-Meier analysis.

**Results:**

Random forest, LASSO, and SVM-RFE identified a low maximum amplitude, decreased albumin level, high creatinine level, increased total bilirubin, and aspartate transaminase (AST) level as risk factors for DIC. Principal component analysis confirmed that these independent variables differentiated between patients who experienced DIC or not, so they were used to construct a nomogram. The nomogram showed good predictive power, with an area under the receiver operating characteristic curve of 0.976 (95% CI 0.948–1.000) and 0.971 (95% CI, 0.914–0.989) in the internal validation. Decision curve analysis indicated clinical utility for the nomogram. DIC was associated with significantly lower 30 days survival for heatstroke patients.

**Conclusion:**

A nomogram incorporating coagulation-related risk factors can predict DIC in patients with heatstroke and may be useful in clinical decision-making.

## 1. Introduction

Heatstroke is a life-threatening illness manifesting as extreme hyperthermia (>40°C), dysfunction in the central nervous system, and multiple organ failure (1, 2). Although the treatment of heatstroke has improved, heatstroke-related deaths are increasing worldwide, which may worsen due to global warming (3, 4).

A substantial proportion of patients with heatstroke, from 22 to 45%, experience disseminated intravascular coagulation (DIC), which further increases risk of multiple organ dysfunction and mortality (5, 6). DIC is difficult to diagnose and challenging to treat. Reliable prediction of which heatstroke patients are at greater risk of DIC could help clinicians monitor such patients more closely and initiate preventive or therapeutic measures earlier. However, the conventional coagulation tests typically used to diagnose DIC are poor predictors of the complication (7).

Several studies have explored potentially better predictors of DIC, such as thromboelastography maximum amplitude, activated clotting time and clot rate as determined with a Sonoclot^@^ device (8–10). However, these biomarkers reflect primarily coagulation, so they may identify patients already in early stages of DIC rather than predict the complication before it occurs. Due to the complex pathogenesis of DIC, a more comprehensive panel of biomarkers may be needed to predict DIC in heatstroke patients.

The current study explored a range of potential risk factors of DIC and selected the best to create a predictive nomogram, which we validated using a 10 years retrospective dataset that included survival at 30 days after admission.

## 2. Materials and methods

### 2.1. Study design and patients

This retrospective study was approved the Ethics Committee of the 908th Hospital of Logistic Support Force (Nanchang, China), which waived the requirement for consent because all participants, at the time of treatment, signed written consent for their anonymized medical data to be analyzed and published for research purposes. All procedures involving human participants were performed in accordance with the 1975 Helsinki Declaration and its later amendments.

We screened for eligibility all patients with heatstroke who were admitted to the intensive care unit of the 908th Hospital from May 2012 to October 2022. Eligible patients had a history of exposure to hot and humid weather or high-intensity activity, and they met at least one of the following criteria based on the Chinese Expert Consensus on the Diagnosis and Treatment of Heatstroke (1): (1) neurological dysfunction, including coma, convulsions, delirium, or abnormal behavior; (2) core temperature ≥ 40°C; (3) functional impairment of at least two organs; or (4) severe coagulopathy or DIC. Severe coagulopathy was defined as the presence of at least two of the following criteria: platelet count < 100,000 cells per μL, international normalized ratio > 1.5, fibrinogen level < 1.50 g/L, and D-dimer value above 10 times the upper limit of normal (11). Patients were excluded if they were younger than 18 years, if they had a congenital coagulopathy or severe chronic disease of the liver or kidney, or if they were using anticoagulant drugs at admission.

Included patients were divided into two groups based on presence or absence of DIC at admission, which was diagnosed based on the scoring system proposed by the International Society of Thrombosis and Hemostasis (12). DIC was diagnosed if a patient had a total score of at least five after summing the points for the following four parameters: platelet count, scored as one point if <100 × 10^9^/L or two points if <50 × 10^9^/L; prothrombin (PT) prolongation time, scored as one point if >3 s, or two points if >6 s; fibrinogen level, scored as one point if <1.0 g/L; fibrin degradation products or D-dimer level, scored as two points if ≥5-fold the upper limit of the normal range, or three points if ≥10-fold the upper limit (13).

### 2.2. Data collection

Baseline clinicodemographic data were extracted from electronic medical records, including age, sex, core temperature (rectal temperature), heart rate, Glasgow coma scale score, and the Acute Physiology and Chronic Health Evaluation II score. Data on the following routine coagulation indicators were collected: PT, activated partial thrombin time, fibrinogen, international normalized ratio, thrombin time, and levels of fibrin degradation product and D-dimer. Data on the following TEG indexes were collected: reaction time (R time), kinetics of clot development (K time), angle, maximum amplitude (MA). Data were collected on the following whole blood characteristics: counts of platelets and red and white blood cells, neutrophil-to-lymphocyte ratio, hemoglobin range and hematocrit percentage. In addition, data were collected on levels of C-reactive protein, alanine transaminase (ALT), AST, total bilirubin (Tbil), albumin, creatinine (Cr), myoglobin, and creatine kinase.

### 2.3. Statistical analysis

All statistical analyses were performed using R 4.2.1 software for windows (Chicago, IL), and all analyses were two-sided. Continuous variables with normal distribution were presented as mean ± standard deviation, while continuous data with a skewed distribution were expressed as median with interquartile range (IQR). Categorical variables were expressed as percentages (%). Pairwise comparisons were conducted using Student’s *t*-test or the Mann-Whitney U test for continuous variables, while the chi-squared test or Fisher’s exact test was used for categorical variables with normal or skewed distributions, as appropriate. Differences were considered significant if *P* < 0.05.

Potential risk factors of DIC were identified using three algorithms: least absolute shrinkage and selection operator (LASSO) regression, support vector machine-recursive feature elimination (SVM-RFE), and random forest. Risk factors identified by all three models were used to construct a nomogram using the *rms* package in R 4.2.1 software for windows. The discriminatory ability of the nomogram was evaluated in terms of areas under receiver operating characteristic curves (AUCs) and calibration curves. Principal component analysis was used to assess the ability of DIC biomarkers. The bootstrapping method (resampling = 500) was used for internal validation. The net benefit rate of the nomogram was assessed using decision curve analysis. Kaplan–Meier curve describing survival at 30 days after admission was compared between patients with or without DIC using the log-rank test.

## 3. Results

### 3.1. Patient characteristics

**FIGURE 1:**
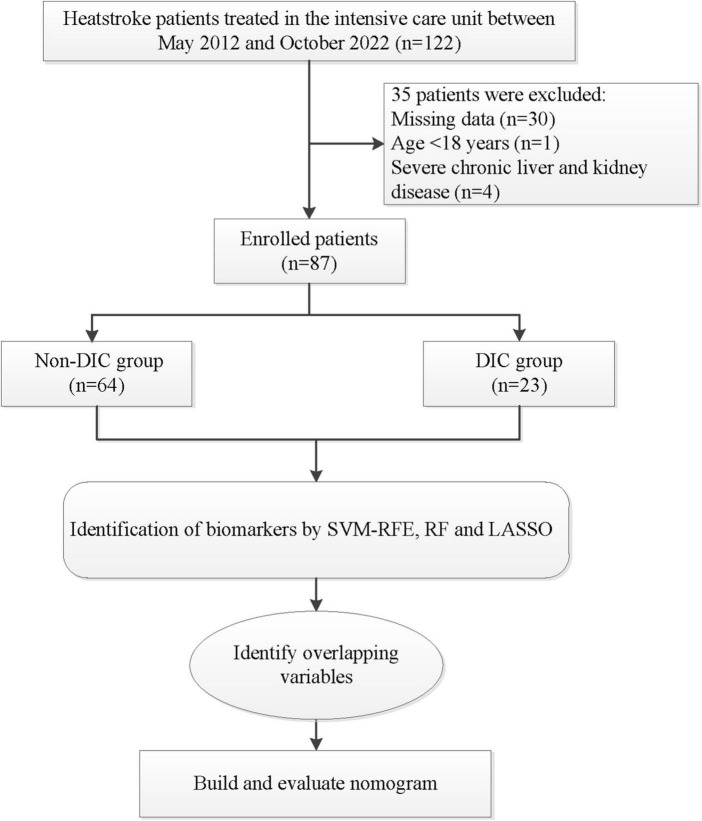
Flowchart of patient selection and analysis. DCA, decision curve analysis; LASSO, least absolute shrinkage and selection operator; RF, random forest; SVM-RFE, support vector machine-recursive feature elimination.

Of the 122 patients considered for enrollment, 87 were included into the final analysis, of whom 23 had DIC, while 64 did not (Figure 1). There were no significant differences between the two groups in terms of sex distribution, age, counts of white or red blood cells, or myoglobin levels (Table 1). Patients with DIC were more likely to have a higher core temperature and increased levels of the following: fibrinogen degradation product, D-dimer, AST, ALT, Tbil, Cr and creatine kinase. As expected, indicators of coagulation were also altered in patients with DIC, reflected by longer R time and K time; longer PT, activated partial thrombin time and thrombin time; and a higher international normalized ratio. Conversely, compared to non-DIC patients, those with DIC had lower levels of angle, MA, fibrinogen, hemoglobin, hematocrit, albumin and platelet count. Overall, DIC patients showed more severe illness and injury to the central nervous system than patients without DIC.

**TABLE 1:** Clinicodemographic characteristics of patients with heatstroke at admission to the intensive care unit, stratified by disseminated intravascular coagulation (DIC) diagnosis.

Characteristics	Total (*n* = 87)	No DIC (*n* = 64)	DIC (*n* = 23)	*P*
Male	76 (87.4)	55 (85.9)	21 (91.3)	0.72
Age, yr	43 (22, 64)	28 (21.8, 64)	48 (40, 61)	0.211
Core temperature, °C	37.6 (36.8, 38.6)	37.4 (36.7, 38.4)	38.4 (37.6, 39)	0.006
R, min	7.4 (5.7, 12.4)	6.5 (5.2, 9.4)	14.9 (10.6, 28.6)	<0.001
K, min	3.2 (2.2, 6.2)	2.6 (2, 4.2)	10.6 (5.9, 22)	<0.001
Angle, °	50.3 (31.4, 60.5)	54.9 (43.8, 62.4)	22 (9.4, 35.1)	<0.001
MA, mm	49.8 (38.4, 56.5)	53.1 (46.3, 59.4)	30.2 (19.5, 40.1)	<0.001
PT, s	16.9 (13.7, 19.8)	14.8 (13.3, 17.4)	27.6 (21.8, 43.5)	<0.001
INR	1.4 (1.2, 1.6)	1.2 (1.1, 1.4)	2.2 (1.8, 3.2)	<0.001
APTT, s	36.1 (28.8, 48.4)	31.6 (28, 40.4)	46.3 (39.8, 97.8)	<0.001
Fibrinogen, g/L	2.0 (1.5, 2.7)	2.2 (1.8, 2.9)	1.4 (1.0, 2.1)	0.002
TT, s	16.8 (14.8, 20.5)	16.4 (14.6, 18.2)	22.0 (16.8, 26.2)	0.002
FDP, μg/L	8.1 (2.2, 26.4)	3.2 (1.2, 9.9)	34.0 (24.5, 65.9)	<0.001
D-dimer, μg/L	2.3 (0.5, 6.7)	1.0 (0.3, 2.7)	8.1 (4.1, 28.1)	<0.001
WBC, ×10^9^/L	11.7 (8, 16.6)	11.4 (7.8, 16.8)	12.5 (8.9, 14.6)	0.733
NLR	10.3 (5.0, 17.4)	10.2 (4.3, 14.7)	11.7 (6.9, 22.6)	0.075
CRP, μg/L	2.4 (0.6, 15.1)	2.2 (0.6, 10.8)	5.7 (1.0, 31.6)	0.192
RBC, ×10^12^/L	4.3 ± 0.7	4.4 ± 0.6	4.1 ± 0.7	0.058
HGB, g/L	133 (118, 143)	136 (121, 145)	119 (109, 133)	0.003
HCT, %	39.4 ± 5.9	40.4 ± 5.3	36.6 ± 6.5	0.018
Platelet, ×10^9^/L	115 (52, 202)	155 (101, 228)	35 (22, 58)	<0.001
AST, U/L	49.1 (21.0, 164.9)	30.4 (18.6, 62.8)	443.1 (98.6, 1105.0)	<0.001
ALT, U/L	75.7 (28.5, 306.0)	41.5 (23.7, 134.4)	687.8 (157.9, 1543.4)	<0.001
Tbil, μmol/L	16.4 (12.3, 25.3)	15.1 (11.3, 19.1)	28.0 (19.3, 78.2)	<0.001
Albumin, g/L	37.9 (34.3, 43.8)	40.0 (36.8, 44.5)	33.2 (25.6, 35.5)	<0.001
Cr, μmol/L	105.9 (77.4, 150.3)	91.0 (72.4, 123.9)	207.9 (113.7, 250.5)	<0.001
MYO, ng/mL	632.9 (118.6,926.9)	445.7 (48.0, 915.1)	834.8 (570.9, 944.5)	0.052
CK, U/L	696 (226, 1975)	402 (192, 1224)	2216 (702, 10179)	<0.001
HR, min^–1^	100 (81, 110)	90 (73, 105)	116 (102, 134)	<0.001
PH	7.4 (7.3, 7.4)	7.4 (7.4, 7.5)	7.3 (7.3, 7.4)	<0.001
PaCO_2_, mmHg	33.5 (28.0, 39.6)	33.8 (28.2, 39.0)	33 (28.1, 42.8)	0.535
PaO_2_, mmHg	145 (86.5, 187)	143.5 (88.6, 182)	158 (82.2, 204.5)	0.765
Lac, mmol/L	2.4 (1.1, 4.9)	1.6 (1.0, 3.2)	4.9 (3.4, 7.6)	<0.001
GCS score	6 (4, 14)	10 (5, 15)	4 (3, 5)	<0.001
APACHE II score	21 (12, 26)	19 (11, 24)	28 (23, 34)	<0.001

Values are *n* (%), mean ± SD, or median (interquartile range), unless otherwise noted. R, reaction time; K, kinetics of clot development; MA, maximum amplitude; PT, prothrombin time; APTT, activated partial thrombin time; INR, international normalized ratio; TT, thrombin time; FDP, fibrinogen degradation product; WBC, white blood cell count; NLR, neutrophil to lymphocyte ratio; CRP, C-reaction protein; RBC, red blood cell count; HGB, hemoglobin; HCT, hematocrit; ALT, alanine transaminase; AST, aspartate transaminase; Tbil, total bilirubin; Cr, creatinine; MYO, myoglobin; CK, creatine kinase; HR, heart rate; Lac, lactate; GCS, glasgow coma scale; APACHE II, acute physiology and chronic health evaluation II.

### 3.2. Identification of DIC biomarkers

**FIGURE 2:**
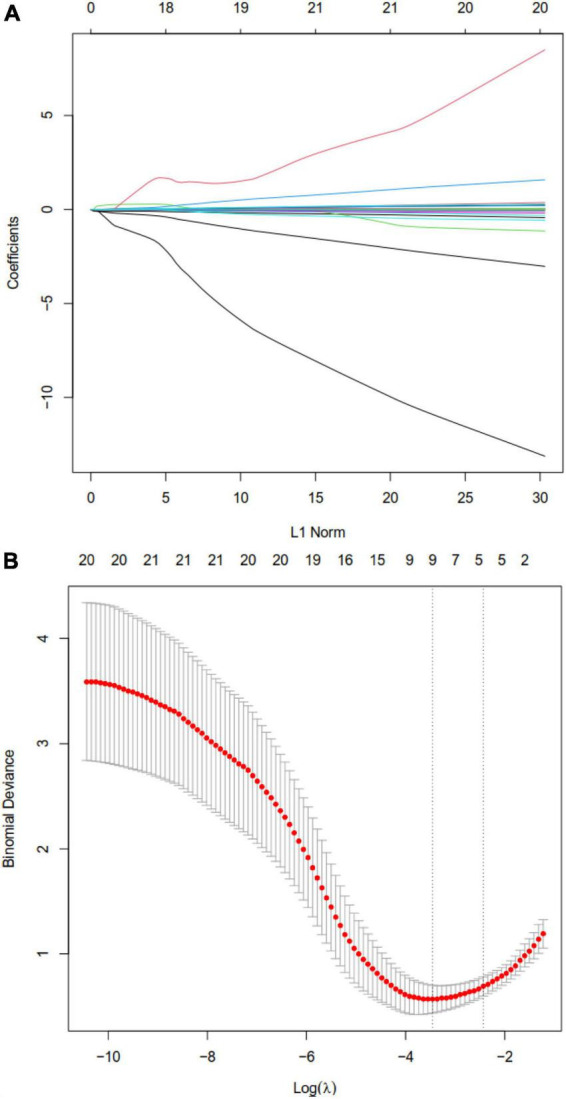
Least absolute shrinkage and selection operator (LASSO) regression to identify clinical features that may predict disseminated intravascular coagulation (DIC) in heatstroke patients. **(A)** LASSO coefficient profiles. **(B)** LASSO regression using 10-fold cross-validation and the “minimum plus one standard error” criterion to identify the optimal penalization coefficient lambda (λ).

**FIGURE 3:**
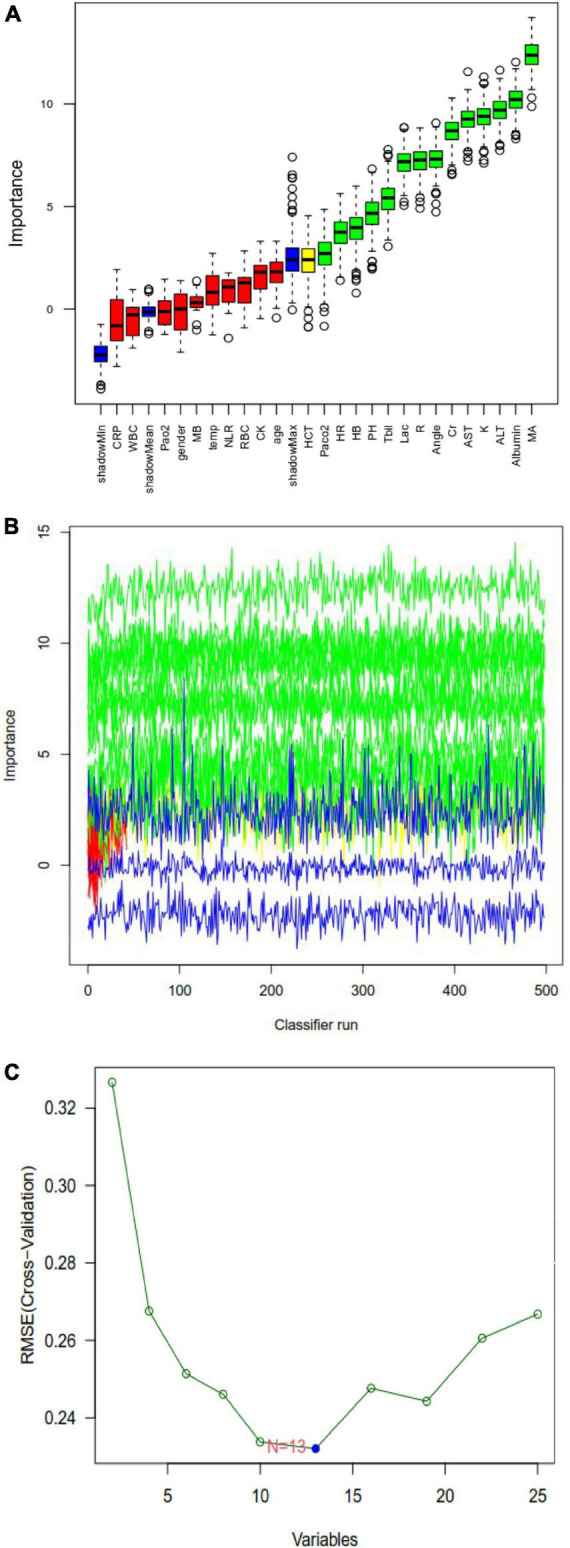
Random forest analysis to identify clinical features that may predict disseminated intravascular coagulation (DIC) in heatstroke patients. **(A)** Boxplot for all features in random forest analysis. Green indicates important variables; red, blue, or yellow, rejected variables. **(B)** Rejection or acceptance of factors during random forest classification runs. **(C)** Support vector machine-recursive feature elimination (SVM-RFE) to identify clinical predictors of DIC.

**FIGURE 4:**
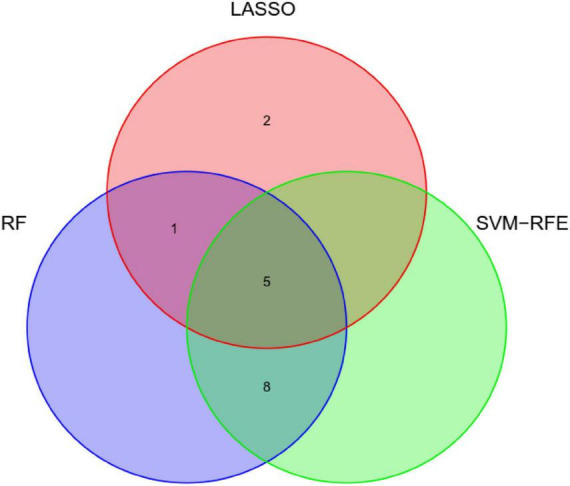
Venn diagram showing overlap of clinical factors that least absolute shrinkage and selection operator (LASSO), random forest, and support vector machine-recursive feature elimination (SVM-RFE) methods identified as predictors of disseminated intravascular coagulation (DIC) in heatstroke patients.

Least absolute shrinkage and selection operator regression analysis identified eight clinical features as potential predictors of DIC in patients with heatstroke: core temperature, ALT, maximum amplitude, hemoglobin, creatinine, albumin, total bilirubin, and creatine kinase (Figure 2). Random forest analysis identified the same six features as LASSO well as the following eight: AST, reaction time, kinetics of clot development, Angle, heart rate, lactate, PaCO_2_, and pH (Figures 3A, B). SVM-RFE identified five of the same features as LASSO and random forest, as well as another eight: AST, reaction time, kinetics of clot development, Angle, heart rate, lactate, PaO_2_, and pH (Figure 3C). The five factors overlapping across all three methods - MA, Cr, Tbil, albumin, and ALT - were considered potential predictors of DIC and used in further analyses (Figure 4).

### 3.3. Verification of DIC biomarkers

**FIGURE 5:**
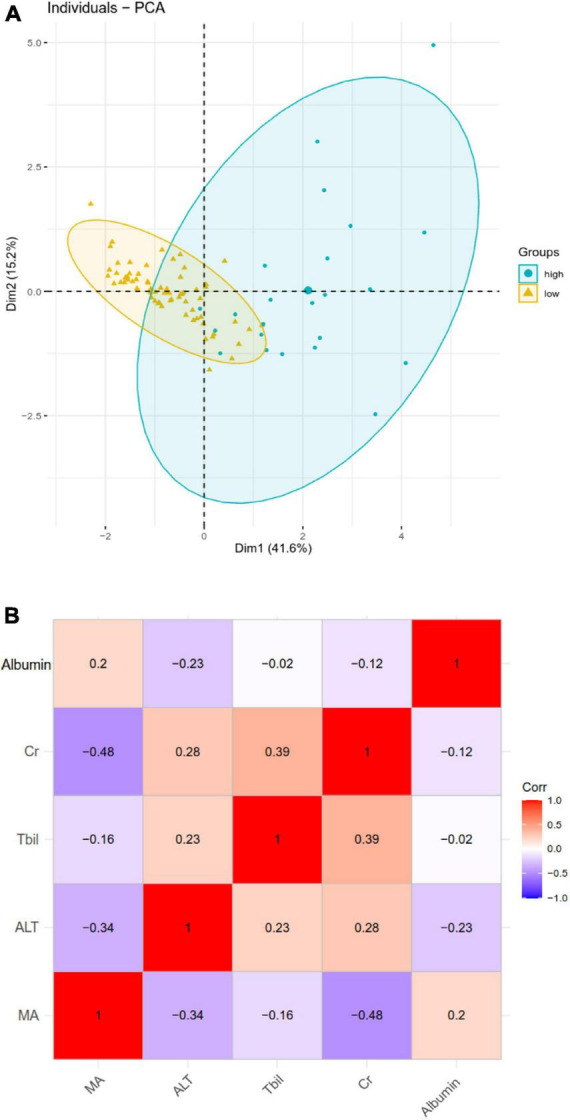
**(A)** Principal component analysis to assess the ability of the five selected variables to differentiate heatstroke patients with or without disseminated intravascular coagulation (DIC). **(B)** Heatmap of pairwise correlations among the five variables. ALT, alanine transaminase; Cr, creatinine; MA, maximum amplitude; Tbil, total bilirubin.

Principal component analysis was used to assess the ability of the five variables identified by LASSO, random forest, and SVM-RFE methods to differentiate patients with or without DIC (Figure 5A). There were no significant correlations among the five variables, suggesting that they had no function similarities (Figure 5B).

### 3.4. Development and validation of a predictive nomogram

**FIGURE 6:**
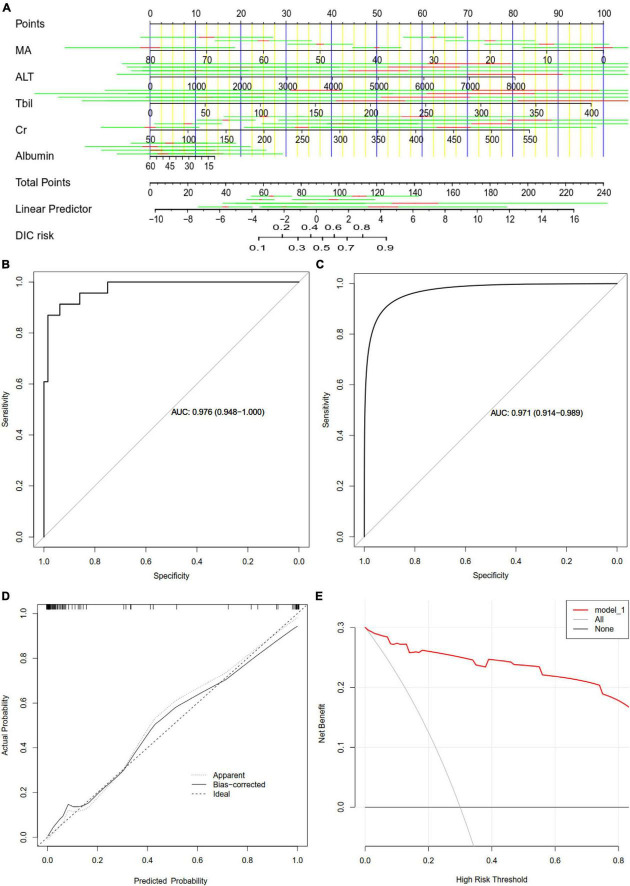
Assessment of a nomogram based on five predictors of disseminated intravascular coagulation (DIC) in patients with heatstroke. **(A)** Nomogram for predicting DIC in patients with heatstroke. **(B)** Receiver operating characteristic curves assessing the ability of the nomogram to predict DIC. **(C)** Internal validation using the bootstrap method (resampling = 500). **(D)** Calibration curve of the predictive model showing the degree of consistency between the predicted probability and actual probability (the Hosmer–Lemeshow test, *P* > 0.05, suggesting that it is of goodness-of-fit). **(E)** Decision curve analysis to assess the clinical benefit of the predictive nomogram. ALT, alanine transaminase; AUC, areas under receiver operating characteristic curves; Cr, creatinine; MA, maximum amplitude; Tbil, total bilirubin.

We developed a nomogram to predict DIC based on the five verified factors (Figure 6A). Our nomogram showed good predictive power, with an AUC of 0.976 (Figure 6B), which was internally validated by bootstrapping, which gave an AUC of 0.971 (Figure 6C). A calibration curve of the predictive model showed a high degree of consistency between the predicted probability and actual probability and confirmed that the nomogram accurately predicted DIC (Figure 6D). Furthermore, decision curve analysis demonstrated that our nomogram had an extensive range of cutoff probabilities and excellent net benefits for threshold probabilities, which showed the potential clinical utility of the predictive model (Figure 6E).

### 3.5. Patient outcomes

**FIGURE 7:**
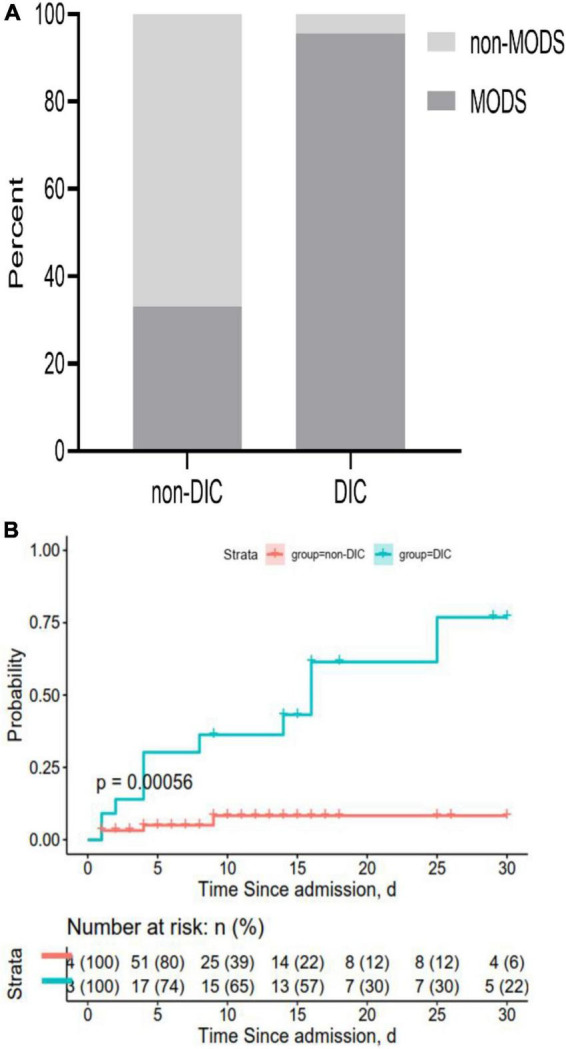
Rates of **(A)** multiple-organ dysfunction syndrome (MODS) and **(B)** overall survival at 30 days after admission. Both panels show data for all 87 patients in the sample.

Across all patients in our study, 95.6% in the DIC group experienced multiple-organ dysfunction by 30 days after admission, compared to only 33.3% in the non-DIC group (*P* < 0.05) (Figure 7A). DIC patients also showed a significantly lower overall survival rate at 30 days (47.8 vs. 6.3%; Figure 7B).

## 4. Discussion

To our knowledge, this article firstly reported a nomogram for prediction of heatstroke induced DIC. Patients suffering from heatstroke are at high risk of developing DIC, which remains a major cause of mortality (14). In this retrospective study, the incidence of DIC was 26.4% and the rate of mortality in patients with DIC was 47.8%. In an effort to predict DIC in order to improve management and timely treatment, we used three complementary methods including SVM-RFE, LASSO and random forest to screen for clinical factors that could reliably predict the complication, and we validated a nomogram for this purpose. SVM is a novel small sample method and a rather robust classification tool. Random forest and lasso can well deal with the high-dimensional data. The resulting model incorporates five routine clinical indexes that are easily acquired within 24 h of hospital admission and that capture complementary aspects of DIC pathophysiology, which may make our nomogram more reliable than other DIC predictors. Result from PCA analysis further showed these variables can clearly distinguished DIC and non-DIC, which indicated that they may play important roles in the prediction of DIC.

Heatstroke directly affects platelet function and can induce organ function damage (15, 16). Previous studies reported that platelet abnormality and hypofibrinogenemia in heatstroke patients increases risk of multiple-organ dysfunction syndrome and heatstroke-induced coagulopathy, with the latter often progressing to DIC (17–19). Therefore, it was not completely surprising that we detected maximum amplitude, a measure of interaction between platelets and fibrinogen used in thromboelastography, as an independent risk factor for DIC (20, 21). We also found that low albumin level, elevated creatinine, high glutamic-pyruvic transaminase, and total bilirubin were positively related to the progression to DIC. Heatstroke patients suffer damage to the liver and kidney, and both organs produce hormones that affect coagulation homeostasis (22–24).

ROC analysis is a traditional method that evaluates the performance of a model (25). The predictive nomogram constructed in our study has a better ability for predicting DIC based on the value of AUC. However, an AUC alone is insufficient to determine that a model has good performance in improving decision-making. DCA and calibration curve were also introduced to estimate the clinical utility and predictive capacity of a nomogram, respectively (26, 27). Results showed the prediction model exhibited acceptable calibration and DCA gave the heatstroke population net benefit of nomogram at different threshold probabilities. Overall, the current predictive model exhibited good performance regarding DIC prediction.

Our model should be further developed and optimized in light of the fact that it is based only on the first 24 h in the intensive care unit, so it does not take into account dynamics in indicator levels. The model was developed with data from patients at a single medical center, so it should be validated in other patient populations.

This work establishes the feasibility of accurately predicting DIC in heatstroke patients on the basis of a few carefully selected clinical variables that are accessible to most medical centers. Our nomogram may become increasingly useful as the incidence of heatstroke increases worldwide.

## Data availability statement

The raw data supporting the conclusions of this article will be made available by the authors, without undue reservation.

## Ethics statement

The studies involving human participants were reviewed and approved by the Ethics Committee of the 908th Hospital of Logistic Support Force. Written informed consent for participation was not required for this study in accordance with the national legislation and the institutional requirements.

## Author contributions

QZ, LZ, LH, QL, and JS: acquisition of data. QZ, LZ, and NZ: statistical analysis. QZ: drafting manuscript. JS: manuscript revision. All authors read and approved the final manuscript.
